# Selenium Attenuates Radiation Colitis by Regulating cGAS‐STING Signaling

**DOI:** 10.1002/advs.202403918

**Published:** 2024-09-30

**Authors:** Qian Xue, Haoqiang Lai, Haimei Zhang, Guizhen Li, Fen Pi, Qifeng Wu, Siwei Liu, Fang Yang, Tianfeng Chen

**Affiliations:** ^1^ Department of Radiation Oncology of Puning People's Hospital Department of Chemistry of Jinan University State Key Laboratory of Bioactive Molecules and Druggalibility Assessment MOE Key Laboratory of Tumor Molecular Biology Jinan University Guangdong China

**Keywords:** cGAS‐STING, DNA damage, radiation colitis, ROS, selenium

## Abstract

Radiation colitis is one of the most common complications in patients undergoing pelvic radiotherapy and there is no effective treatment in the clinic. Therefore, searching for effective agents for the treatment of radiation colitis is urgently needed. Herein, it is found that the essential element selenium (Se) is protective against radiation colitis through inhibiting X‐ray‐induced apoptosis, cell cycle arrest, and inflammation with the involvement of balancing the generation of reactive oxygen species after the irradiation. Mechanistically, Se, especially for selenium nanoparticles (SeNPs), induced selenoprotein expression and then functioned to effectively restrain DNA damage response, which reduced X‐ray‐induced intestinal injury. Additionally, SeNPs treatment also restrained the cyclic GMP‐AMP synthas (cGAS)‐ stimulator of interferon genes (STING)‐TBK1‐IRF3 signaling pathway cascade, thereby blocking the transcription of inflammatory cytokine gene, IL‐6 and TNF‐α, and thus alleviating inflammation. Moreover, inducing selenoprotein expression, such as GPX4, with SeNPs in vivo can regulate intestinal microenvironment immunity and gut microbiota to attenuate radiation‐induced colitis by inhibiting oxidative stress and maintaining microenvironment immunity homeostasis. Together, these results unravel a previously unidentified modulation role that SeNPs restrained radiation colitis with the involvement of inducing selenoprotein expression but suppressing cGAS‐STING‐TBK1‐IRF3 cascade.

## Graphical abstract

In this study, it is found that SeNPs play a regulatory role in restricting X‐ray‐induced intestinal tissue damage and the inflammation response with the involvement of increasing selenoprotein expression and decreasing DNA damage response as well as the cGAS‐STING pathway cascade, which may provide insight into the use of Se in radiation colitis treatment.

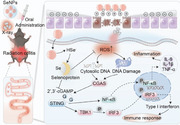

## 1 Introduction

As one of the three major conventional cancer treatments, radiotherapy is often the first choice of adjuvant therapy because it kills tumor cells directly with high‐energy X‐rays. According to statistics, more than 70% of cancer patients receive radiotherapy in the course of treatment.^[^ ^1^ ^]^ However, the use of X‐ray irradiation in radiotherapy inevitably causes damage to some normal cells or tissues that are sensitive to radiation. Despite advancements in technology and the development of precision radiotherapy, up to 90% of patients with pelvic, abdominal, and colorectal tumors still experience gastrointestinal reactions or discomfort shortly after radiotherapy.^[^ ^2^ ^]^ Common symptoms include nausea, vomiting, diarrhea, weight loss, and rectal bleeding, which in severe cases can lead to death.^[^ ^3^ ^]^ Therefore, searching for strategies that could inhibit or attenuate radiation‐induced damage to intestinal tissue and the subsequent inflammation response is of critical importance for cancer treatment, especially for pelvic cancer treatment.

Although pathologically similar to inflammatory bowel disease (IBD), the incidence of radiation colitis is much higher than that of IBD.^[^ ^4^ ^]^ The pathogenesis of radiation colitis is mainly due to radiation‐induced hydrolysis producing reactive oxygen species (ROS), such as hydrogen peroxide, superoxide anion radicals, and hydroxyl radicals, which triggers a variety of pathways associated with intestinal epithelial cell death^[^ ^5^ ^]^ and thus disrupts the intestinal mucosal barrier and subsequently results in inflammation and bleeding.^[^ ^6^ ^]^ Studies have revealed that the primary mechanism of radiation‐induced intestinal injury is correlated with the rapid death of mitotic cells,^[^ ^7^ ^]^ apoptosis,^[^ ^8^ ^]^ and some other mechanisms.^[^ ^9^ ^]^ Strategies that target and modulate these mechanisms have also been postulated, but the outlook is not promising from a clinical translation and practical point of view, necessitating the development of new treatments for radiation colitis.

The innate immune system in the human body, serving as the first wall of defense against invading pathogens, has evolved a sensing mechanism capable of recognizing and eliminating potential pathogens. This is primarily achieved by detecting pathogen‐associated molecular patterns (PAMPs) and damage‐associated molecular patterns (DAMPs), to defend against microbial intrusion.^[^ ^10^ ^]^ The roles of the cyclic GMP‐AMP synthetase (cGAS) and interferon gene‐stimulating protein (STING) pathways in the detection of master of ceremonies and exogenous double‐stranded DNA (dsDNA), along with bacterial production of cyclic dinucleotides (CDNs), have been considered to play important roles in inducing inflammation response. Numerous investigations have confirmed that the cGAS‐STING signaling pathway is significantly associated with many immune‐related diseases and is very important for intestinal health.^[^ ^11^ ^]^ Activation of the cGAS‐STING signaling pathway is linked with different kinds of cell death^[^ ^11^, ^12^ ^]^ and is important in defending against viruses and resisting bacterial invasion.^[^ ^13^ ^]^ Additionally, the cGAS‐STING signaling persistence cascade may exacerbate intestinal inflammation, disrupting the integrity of the intestinal structure and the balance of the environment^[^ ^14^ ^]^ as well as infection prevention.^[^ ^15^ ^]^ Therefore inhibition of this pathway may help to maintain the homeostasis of the intracellular environment.

Since the gut environment is complex, the incidence and progression of radiation enteritis are very complicated. The interaction between intestinal epithelial cells, immune cells, and microbiota is the basis for maintaining intestinal homeostasis. Radiotherapy could induce overproduction of ROS, which induces DNA damage to intestinal cells leading to cell death, thereby destroying the wholeness of the intestinal barrier, which may facilitate the penetration of bacteria or gut microbiome into the gut from the lumen, contributing to enlist and enactment of immune cells in the lamina propria, further exacerbating the inflammatory response process. Emerging evidence has disclosed the roles of the gut microbiome in radiation colitis. Radiation causes changes in the assortment and variety of intestinal flora, mainly manifested in the decrease of colonization of probiotics such as *Lactobacilli* and *Bifidobacteria*, which may enhance the proliferation of pathogenic bacteria such as *Proteus*, *gamma Proteobacteria*, and *Coprococcus*, and then increase of intestinal permeability, and finally lead to the occurrence of radiation enteritis.^[^ ^16^ ^]^ Fecal bacteria transplantation and probiotics/probiotics intervention can alleviate the radiation damage, which may represent an effective strategy to treat or prevent radiation enteritis.^[^ ^17^ ^]^ Evidence has also demonstrated the benefit of regulating the gut microbiome to alleviate enteritis.^[^ ^18^ ^]^ Therefore, searching for agents that possess high efficiency and low toxicity for modulating or remodeling the diversity of gut microbiome may help for radiation colitis treatment.

Selenium (Se) is an essential element for humans.^[^ ^19^ ^]^ It takes on a role in growth, advancement, and various physiological processes, primarily through the regulation of selenoproteins.^[^ ^20^ ^]^ Research indicates that Se deficiency can lead to oxidative stress and various chronic diseases associated with inflammation.^[^ ^20^, ^21^ ^]^ Preliminary clinical findings suggest that Se deficiency is common in patients with inflammatory bowel disease. This deficiency can exacerbate the severity of intestinal damage and inflammatory responses.^[^ ^22^ ^]^ 25 selenoproteins in the human body play important roles in inhibiting inflammatory responses, alleviating oxidative stress, and regulating and supporting protective gut flora.^[^ ^23^ ^]^ GPX2 and GPX3 were confirmed to promote the self‐renewal ability of the intestinal epithelium cells.^[^ ^24^ ^]^ Evidence has also disclosed the regulation roles of Se in the diversity of gut microbiota.^[^ ^20a^ ^]^ However, the biological functions of Se are dependent on its chemical form and which Se species possess better protective effects on intestinal damage remains elusive. Therefore, searching for novel Se ‐based agents with high efficiency and low toxicity for the powerful prevention or treatment of intestinal inflammatory diseases is a topic of intense research.

**Scheme 1:**
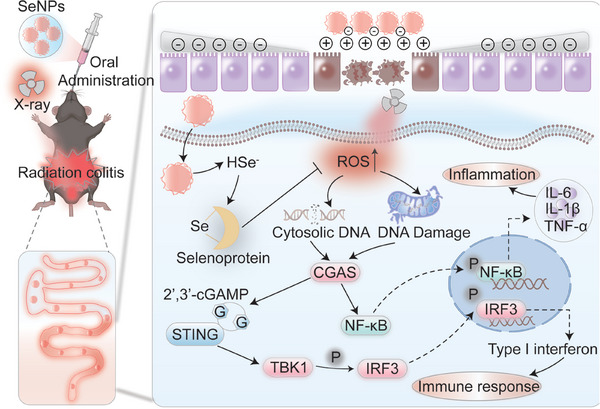
Schematic illustration of Se in antagonizing X‐ray‐induced colitis.

In this paper, after confirming the low toxicity of several different forms of selenium to normal intestinal epithelial cells and normal intestinal crypt epithelial cells, we compared the protective outcome of these selenium compounds against radiation‐induced damage to intestinal epithelial cells in the cellular model at an optimal radiation damage dose. Through screening, we found that selenium nanoparticles (SeNPs) are characterized by low toxicity, high biotransformation rate, and better resistance to radiation damage to intestinal epithelial cells compared to other forms of selenium. We also observed that SeNPs could effectively restrict X‐ray‐induced ROS overproduction and the subsequent DNA damage. Additionally, SeNPs may upregulate GPX4 expression upon X‐ray irradiation stress and alleviate X‐ray‐triggered cGAS‐STING‐IRF3/NF‐κB signaling cascade activation in vitro. Furthermore, SeNPs pretreatment enhanced GPX4 expression in the intestinal tissue and reduced the damage effects and the inflammation response induced by X‐ray with the involvement of inhibiting DNA damage and suppressing STING expression. SeNPs were also found to restrict the disruption induced by X‐ray in the intestinal immune microenvironment by facilitating the proportion of M2 macrophages and neutrophils, which cooperated to effectively intervene the radiation‐induced colitis (**Scheme** **1**).

## 2 Results and Discussion

### 2.1 Se Reversed X‐Ray‐Induced Damages to Intestinal Epithelial Cells Through Maintaining Mitochondria Homeostasis

**Figure 1:**
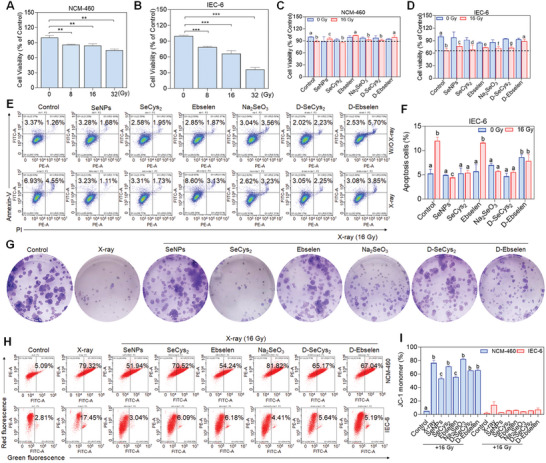
Protective effect of selenium compounds on IEC‐6 cells and NCM‐460 cells against X‐rays. A,B) The survival rate of NCM‐460 and IEC‐6 cells after 48 h of X‐ray irradiation (0, 8, 16 and 32 Gy). Effects of X‐ray (16 Gy) and Se (2 µM) on the cell viability of NCM‐460 cells C) and IEC‐6 cells D). Cells were primed with Se for 6 h followed by X‐ray irradiation. After 48 h, cell viability was scrutinized by MTT assay (Data are represented as mean ± SD, n = 3). E‐F) Effects of Se and X‐ray irradiation on the cell apoptosis on IEC‐6 cells. Data are represented as mean ± SD, n = 2. Bars with characters a, b, and c are denoted as significant differences between the treatment and control groups. G) The colony formation of IEC‐6 cells after the therapy for Se (2 µM) and X‐ray (16 Gy). H‐I) Consequences of Se (2 µM) and X‐ray (16 Gy) on the mitochondria membrane potential of NCM‐460 and IEC‐6 cells. Cells were pre‐exposed to Se for 6 h and then irradiated by X‐ray. After 48 h, cells were assembled and stained with a JC‐1 probe and then analyzed by Flow cytometry assay. Data are represented as mean ± SD, n = 2. **P < 0.01 and **P < 0.001 levels are considered as significant differences in comparison with the untreated control group. Bars with characters a, b and c are denoted as significant differences between the treatment and control groups.

To investigate whether Se could protect intestinal epithelial cells from radiation injury, we first evaluated the proliferation suppression effects of human normal colon mucosal epithelial cells (NCM‐460) and normal intestinal epithelial cells (IEC‐6) after X‐ray irradiation. According to **Figure** **1**A,B, X‐ray treatment inhibited NCM‐460 and IEC‐6 cell proliferation in a dose‐dependent pattern with IEC‐6 cells exhibiting more sensitivity to the irradiation. For instance, upon exposure to X‐rays (16 Gy), the survival rates of NCM‐460 and IEC‐6 cells decreased to ≈84.19% ± 3.7 and 66.42% ± 2.8 when compared to the untreated cells, respectively. When the irradiation dosage was raised to 32 Gy, the cell viability decreased to 74.8% ± 2.4 and 36.03% ± 3.9. As illustrated in Figure 1C,D, Se pretreatment restrained the suppression effects of X‐ray on NCM‐460 cells and IEC‐6 cells. Among the selected Se species, we found that SeNPs, Ebselen and D‐Ebselen preincubation exhibited higher protective effectives against X‐ray irradiation in IEC‐6 cells, which was reflected in the increased cell viability compared to the X‐ray treated group alone. Additionally, Se pretreatment blocked X‐ray‐induced cell apoptosis as evidenced by the decreased apoptotic cell population. For instance, there was 12.00% of apoptotic cells were found in the X‐ray group in IEC‐6 cells, however, the proportion of apoptosis cells was 4.43% and 5.40% in SeNPs and SeCys_2_ preincubation followed by X‐ray treatment groups, respectively (Figure 1E,F), which suggested the protective roles of SeNPs and SeCys_2_ in IEC‐6 cells against irradiation. Additionally, Se pretreatment, especially for SeNPs, could reverse X‐ray‐induced damage to IEC‐6 cells and promote larger colony formation (Figure 1G). Additionally, we also examined whether Se treatment could scale down the antitumor consequences induced by X‐ray.^[^ ^25^ ^]^ As illustrated in Figure S2 (Supporting Information), SeNPs pretreatment did not affect the anti‐tumor capabilities of X‐ray on the cervical carcinoma cell lines such as C33a, SiHa, and Hela, as well as the colon cancer cells CT26, suggesting that the reversal of X‐rays‐induced intestinal injury by Se may not affect the anticancer activity of X‐rays.^[^ ^26^ ^]^ We will further use the tumor model to authenticate this conclusion in our future work. Together, these consequences suggest that Se treatment was managed to turn around the cell growth quelling effects induced by X‐ray. Mitochondria plays a central role in modulating cell death.

To probe the duties of Se in reversing X‐ray‐induced injury to intestinal epithelial cells, we examined the mitochondria potential after Se and X‐ray treatment. As depicted in Figure 1H,I, treatment with X‐rays alone resulted in a 76.65% and 14.06% decrease in mitochondrial membrane potential for NCM‐460 and IEC‐6 cells, respectively. However, Se pretreatment inhibited the damage effects on mitochondria induced by X‐ray, as shown by the lower mitochondrial potential than irradiation groups. Especially, SeNPs exhibited more potent protecting effects against X‐ray irradiation with the depolarized mitochondria in NCM‐460 and IEC‐6 cells decreased to 53.47% and 3.01%, respectively (Figure 1H,I). All the above results demonstrate the protective role of Se against irradiation in intestinal epithelial cells through maintaining mitochondria homeostasis.

### 2.2 Se Effectively Restrained X‐Ray‐Induced ROS Generation and DNA Damage Response

**Figure 2:**
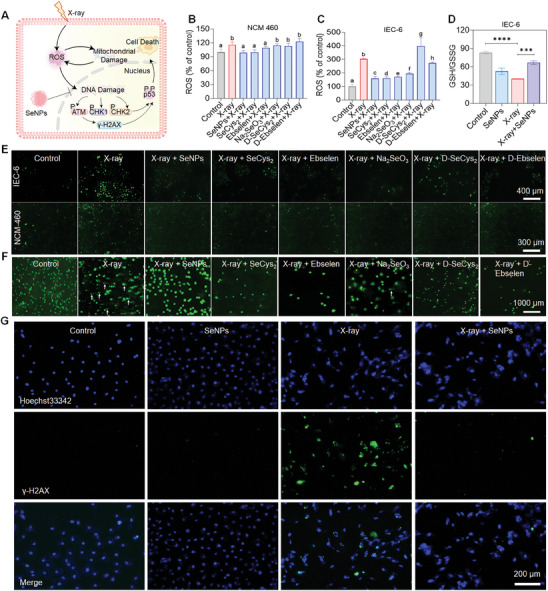
Protective effects of selenium drugs against X‐ray‐induced mitochondrial dysfunction in NCM‐460 and IEC‐6 cells. A) Schematic illustration of SeNPs scavenging ROS in normal small intestinal epithelial cells induced by radiotherapy. Detection of ROS in NCM‐460 B) and IEC‐6 C) cells pretreated for 6 h with different selenium drugs (SeNPs, SeCys_2_, Ebselen, Na_2_SeO_3_, D‐SeCys_2_, and D‐Ebselen) under X‐ray irradiation (16 Gy). Each value represents the mean ± SD of three replicates. Letters a, b, c, d, e, f, g, and h are considered statistically significant at *P* < 0.05. ***P < 0.001 is considered as significant difference between the comparing groups. D) Changes in glutathione expression levels in IEC‐6 cells pretreated with SeNPs after irradiation. E) DCF fluorescence images (green) of NCM‐460 and IEC‐6 cells pretreated for 6 h with different selenium drugs under X‐ray irradiation (16 Gy). F) Representative fluorescence images of DNA double‐strand damage repair in IEC‐6 cells pretreated with nano selenium and irradiated (16 Gy). Scale bar = 1000 µm. The arrow indicates the DNA tails in the comet assay. G) Immunofluorescence analysis of phosphorylated γ‐H2AX, with Hoechst 33342 marked as nuclei (blue) and phosphorylated γ‐H2AX marked in green. Scale bar = 200 µm.

We examined the intracellular location of SeNPs in IECs by fluorescence microscopy. SeNPs entered and located in the lysosome after 1 h treatment and its accumulation in lysosomes became more obvious with the supplement of drug incubation time. We also evaluated the absorption mechanism of SeNPs by IEC6 cells, and the results showed that SeNPs mainly passed through the membrane by endocytosis and were located in the lysosome (Figure S3, Supporting Information). Additionally, to evaluate the endocytosis mechanism, different endocytosis inhibitors (NaN_3_, Dynasore, Sucrose, Chlorpromazine and Nystatin) were used for the evaluation. We found that 4 °C and NaN_3_ pretreatment significantly inhibited the uptake of SeNPs, which suggests that SeNPs enters the cell through the energy‐dependent endocytosis pathway. Additionally, dynasore and sucrose pretreatment significantly decreased the uptake of SeNPs in IEC‐6 cells. These results suggest that SeNPs was absorbed by IEC‐6 cells mainly by dynamin‐mediated lipid raft endocytosis and clathrin‐mediated endocytosis (Figure S4, Supporting Information). ROS overproduction‐mediated DNA damage and mitochondria dysfunction have been verified to a key component of the primary mechanisms of X‐ray.^[^ ^27^ ^]^ To further evaluate whether Se exhibits the protective effects on intestinal epithelial cells against X‐ray was correlated with restricting ROS generation, the intracellular ROS was examined (**Figure** **2**A). X‐ray irradiation‐induced ROS accumulation in NCM‐460 and IEC‐6 cells, while Se pretreatment significantly inhibited the induction of X‐rays,^[^ ^28^ ^]^ with SeNPs having a stronger inhibitory effect (Figure 2B,C), which may be due to the modulation roles of Se on the intracellular reductase system as indicated by the upregulated proportion of GSH/GSSG in the joint treatment groups of SeNPs and X‐ray. The slight reduction of GSH/GSSG may be the result of SeNPs metabolized into Se^−2^ and then HSe^−^ intracellular by using GSH (Figure 2D). These results were further verified by the decreased fluorescence of DCF in NCM‐460 and IEC‐6 cells after cells were pretreated with Se upon X‐ray exposure (Figure 2E; Figure S5A,B, Supporting Information). Furthermore, Se pretreatment inhibited X‐ray‐triggered DNA damage. For instance, X‐ray irradiation induced obvious DNA fragmentation as indicated by the long DNA fluorescence in the tail.^[^ ^29^ ^]^ However, Se preincubation, especially for SeNPs, dramatically inhibited the damage effects as demonstrated by the comet tail, and the DNA fragmentation was less inhibited by Na_2_SeO_3_ incubation. To further estimate the protection of intestinal epithelial cells by Se, we also investigated the expression of histone, one representative hallmark of DNA damage response, after X‐ray treatment. As depicted in Figure 2F, SeNPs pretreatment dramatically restricted the X‐ray‐induced DNA break frequencies as evidenced by the decreased expression of γ‐H2AX (Figure 2G; Figure S5C, Supporting Information). Additionally, we also found the potent antioxidant N‐acetylcysteine (NAC) could inhibit the X‐ray‐induced damage effects, as evidenced by the declined expression of γ‐H2AX in IEC‐6 cells (Figure S6, Supporting Information), which suggests that X‐ray irradiation induces ROS generation and results in DNA damage. These results suggest that Se is effective in reducing X‐ray‐induced DNA damage in the presence of scavenging ROS.

### 2.3 SeNPs Alleviated DNA Damage‐Mediated cGAS‐STING Signaling Pathway Activation Through Inducing Selenoprotein Expression

**Figure 3:**
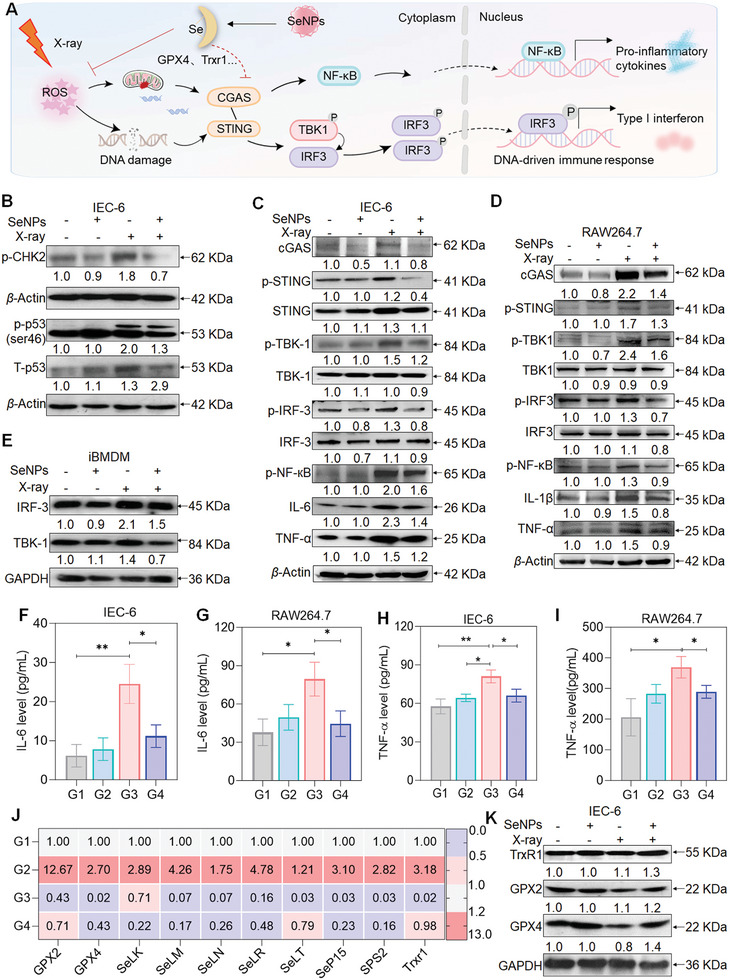
SeNPs suppressed cGAS‐STING signaling pathway activation by inducing selenoprotein expression. A) Schematic diagram of the regulatory mechanism of SeNPs inhibited cGAS‐STING signal cascade under DNA damage response. B) SeNPs inhibited X‐ray‐induced DNA damage in IEC‐6 cells. Effects of SeNPs on cGAS‐STING signaling pathway in IEC‐6 cells C), RAW264.7 cells D) and BMDMs E) upon X‐ray irradiation. Cells were pretreated with SeNPs (2 µM) for 6 h followed by X‐ray (16 Gy) irradiation and 48 h later the total protein was collected and subjected for the analysis of protein expression. F‐I) Expression of TNF‐α and IL‐6 in IEC‐6 and RAW264.7 cells was examined by ELISA assay. Data are represented as mean ± SD, n = 3. J) The mRNA level of selenoprotein in IEC‐6 cells after the treatment of SeNPs (2 µM) and X‐ray (16 Gy) (n = 3). K) Expression of GXP2, GPX4, and TrxR1 in IEC‐6 cells. Cells were pretreated with SeNPs (2 µM) for 6 h and then irradiated by X‐ray (16 Gy). After 48 h, the total protein was collected and submitted to a Western blotting assay. G1: Control, G2: SeNPs, G3: X‐ray, G4: X‐ray + SeNPs. *P < 0.05 and **P < 0.01 are considered statistically significant differences.

The cyclic guanosine phosphate‐cGAS is considered to be an important cytoplasmic DNA receptor in mammalian cells, which can bind double‐stranded DNA to activate STING and induce the demonstration of type I interferon and other inflammatory factors.^[^ ^30^ ^]^ Given the important effect of the cGAS‐STING pathway on inflammatory diseases, we examined whether Se alleviated X‐ray‐triggered DNA damage response could block the cGAS‐STING cascade and suppress inflammation response (**Figure** **3**A). X‐ray treatment induced the upregulation of proteins that are involved in DNA damage including phospho‐CHK2, and phospho‐p53. However, cells treated with SeNPs reversed the inducing capacity of X‐ray as indicated by the downregulated protein expressions, which further confirmed that SeNPs could effectively restrict irradiation‐damaged DNA (Figure 3B). Additionally, we also found that exposure of IEC‐6 cells to X‐rays activated the cGAS‐STING signaling pathway. The demonstration of phospho‐STING, phosphor‐TBK1 and phospho‐IRF3 was upregulated upon irradiation but decreased by pretreatment with SeNPs (Figure 3C). cGAS‐STING pathway activation can stimulate NF‐κB transcription and induce the expression of inflammatory cytokines such as IL‐6, IL‐β and TNF‐α.^[^ ^31^ ^]^ SeNPs preincubation induced downregulated expression of these molecules after the irradiation of X‐ray. Similar results were also found in macrophages including RAW264.7 and the bone marrow‐derived macrophages by Western blot and Elisa assay (Figure 3D–I). These results suggest that SeNPs treatment alleviated X‐ray‐induced DNA‐sensing cGAS–STING pathway activation thus suppressing inflammatory response.

To further investigate how SeNPs blocked the cascade of cGAS–STING, we examined the mRNA expression of selenoproteins. X‐ray irradiation induced a marked reduction in selenoprotein expression including GPX2, GPX4, TrxR1, SeLK, SeLS, SeLM, SeLN, SeLT, and SeP15. However, the SeNPs treatment increased the levels of these selenoproteins and the Western blot assay also confirmed these results as evidenced by the decreased expression of GPX2 and GPX4 under X‐ray treatment but upregulated at the presence of SeNPs (Figure 3J,K). Collectively, these consequences proffer that Se effectively inhibits DNA damage‐mediated activation of the cGAS‐STING pathway, which suppresses X‐ray‐induced inflammation and is involved in the induction of selenoprotein expression.

### 2.4 SeNPs Attenuates Radiation‐Induced Intestinal Damage In Vivo

**Figure 4:**
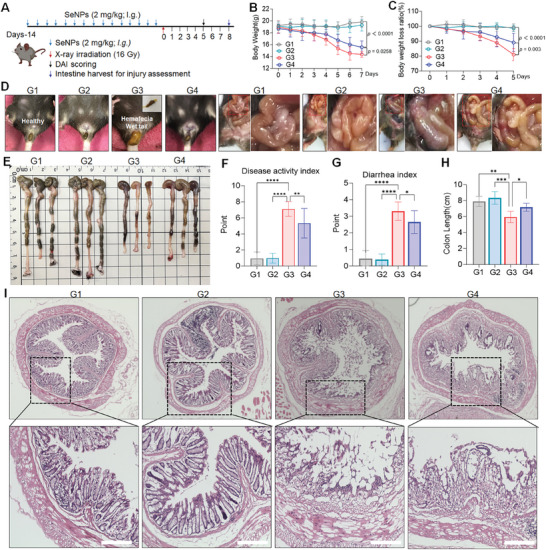
The protective effects of SeNPs against X‐ray radiation therapy on the intestine in vivo. A) Schematic diagram of in vivo radiation protection assessment. B‐C) Changes and loss in mouse body weight (n = 10). D) Representative images of the wet tail and hematochezia on day 5 and intestinal integrity structure on day 8 for each group. E) Representative images of colon length after the treatments. F) DAI for radiation‐induced colitis in each group. G) Diarrhea index (n = 10). H) Quantification of colon length in different treatment groups (n = 3). I) H&E staining images of colon tissues from different treatment groups (scale bar = 400 µm). G1: Control, G2: SeNPs, G3: X‐ray, G4: X‐ray + SeNPs. Each value is represented as mean ± SD. *P < 0.05, **P < 0.01, ***P < 0.001, and ****P < 0.0001 are denoted as significant differences between the comparing groups.

Inspired by the radioprotective properties of SeNPs in vitro, we further evaluated their protective effects against radiation‐induced colitis in C57BL/6J mice in vivo. Previously, we found that SeNPs (2 mg kg^−1^) administration exhibited better protection against cisplatin‐induced nephrotoxicity with a higher safety index. Additionally, we also performed preliminary experiments to investigate the SeNPs effects on X‐ray irradiation and we found that oral administration with SeNPs 2 mg kg^−1^ per day for 14 days possesses better protective effects against X‐ray‐induced injury to colon tissues when compared to 1 mg kg^−1^ dosage (data not shown). Therefore, mice were administered SeNPs (2 mg kg^−1^) every other day for 14 days, with the last dose given 6 h before abdominal irradiation at 16 Gy. Subsequently, hematochezia and histopathological examinations were carried out at specified time points post‐irradiation (**Figure** **4**A). Mice in the radiation‐induced colitis model exhibited signs of colitis, such as significant diarrhea (wet tail) and hematochezia (fecal occult blood), which may account for the acute inflammation response after irradiation. However, SeNPs pretreatment significantly reduced the damage response induced by X‐ray as showed by the weight changes, less weight loss, and less hematochezia (Figure 4B,C). Additionally, we also observed that X‐ray treatment induced more obvious congestion and lower Disease Activity Index (DAI), the symptoms scoring index, while SeNPs treatment attenuated the damages induced by X‐ray including weight loss, colon shortening and higher DAI (Figure 4D–H). Hematoxylin and eoxin (H&E) staining assay further illustrated the protective effect of SeNPs against radiation‐induced colonic damage. As depicted in Figure 4I, the colonic tissue in the irradiation test group suffered severe damage to the villous structure, whereas SeNPs treatment significantly protected the colonic mucosa as evidenced by the villous structures and integrity. Additionally, we also found that SeNPs pretreatment could alleviate the damage effects of X‐ray on the duodenum and ileum tissues (Figure S7, Supporting Information). Together, these data demonstrate that SeNPs pretreatment significantly attenuates X‐ray‐induced intestinal tissues damage.

### 2.5 SeNPs Pretreatment Shapes the Immune Microenvironment of X‐Ray‐Induced Colitis

**Figure 5:**
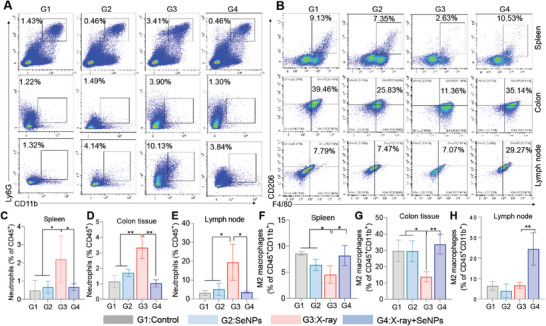
SeNPs inhibit X‐ray‐induced disruption of the intestinal immune microenvironment. Changes of neutrophils A) and M2 macrophages B) in the spleen, colon, and lymph node after the treatment of SeNPs and X‐ray. Mice were pretreated with 2 mg kg^−1^ SeNPs for two weeks and then received with or without X‐ray irradiation (16 Gy). After 10 days, mice were euthanized and the spleen, colon, and lymph nodes were assembled for the analysis of neutrophils and M2 macrophages using a flow cytometry assay. Quantitative analysis of neutrophils C‐E) and anti‐inflammatory M2 polarized macrophages F‐H) in the spleen, colon tissues, and lymph nodes of mice from different treatment groups. G1: Control, G2: SeNPs, G3: X‐ray, G4: X‐ray + SeNPs. Each value is represented as mean ± SD (the sample size was 5, 4, and 3 in spleen, colon tissues and lymph nodes, respectively). *P < 0.05 and **P < 0.01 are denoted as significant differences between the comparing groups.

X‐ray irradiation may disrupt the homeostasis of the colonic immune microenvironment by promoting infiltration of neutrophils and inflammatory macrophages, which thus promotes inflammation response.^[^ ^32^ ^]^ Macrophages and neutrophils exhibit higher resistance levels to radiation‐induced cell death, and studies have shown that M2‐like macrophages possess more resistance to radiation than M1‐like macrophages.^[^ ^33^ ^]^ Since SeNPs pretreatment could inhibit X‐ray‐induced injury in colon tissue, we next examined the changes in immune cell proportion after the treatment of SeNPs and X‐rays to verify the protection roles of SeNPs. As depicted in **Figure** **5**, treatment with SeNPs inhibited radiation‐induced neutrophil infiltration and exacerbation of inflammatory responses and significantly enhanced the proportion of anti‐inflammatory M2 polarized macrophages in the spleen, colon and lymph node tissues compared to the untreated group. Together these effects recommendation that pretreatment with SeNPs significantly inhibits the occurrence of radiation‐induced intestinal inflammatory responses and maintains the homeostasis of the intestine microenvironment immune system to combat inflammatory responses.

### 2.6 SeNPs Inhibits X‐Ray‐Induced Gut Inflammation Through Triggering Selenoprotein Expression

**Figure 6:**
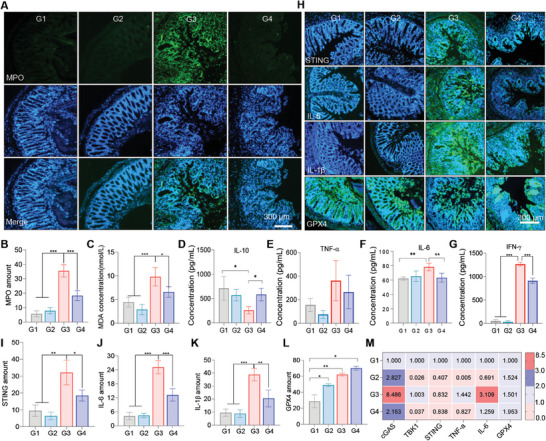
SeNPs inhibited X‐ray‐induced intestine injury and inflammation with the involvement of inactivating cGAS‐STING pathway in vivo. A) Representative images of MPO in different treatment groups (scale bar = 300 µm). B) Semi‐quantitative analysis of MPO in the colon tissues (n = 3). C) Levels of MDA in the colon tissues after different treatments (n = 5). D–G) Levels of IL‐10, TNF‐α, IL‐6, and IFN‐γ in the supernatant of mesenteric lymph node tissues in each group (n = 3). H‐L) Gene manifestation of STING, IL‐6, IL‐1β, and GPX4 in the colon tissues after different treatments (scale bar = 300 µm) (n = 3). M) The mRNA expression of cGAS, STING, TBK1, TNF‐α, IL‐6, and GPX4 in the colon tissues after different treatments (n = 3). G1: Control, G2: SeNPs, G3: X‐ray, G4: X‐ray + SeNPs. Each value represents as mean ± SD of three replicates. *P < 0.05, **P < 0.01, and ***P < 0.001 are denoted as significant differences between the comparing groups.

Since SeNPs treatment could restrict X‐ray‐induced injury in the intestine in vivo, we next evaluated whether SeNPs treatment directly reduces X‐ray‐induced inflammation. As depicted in **Figure** **6**A,B, X‐ray irradiation induced significant upregulation of myeloperoxidase (MPO), the biomarker of activated neutrophils, in the colon tissue as indicated by the enhanced green fluorescence of MPO, which suggests that X‐ray treatment may facilitate the activation of neutrophils. However, SeNPs pretreatment significantly suppresses the expression of MPO in colon tissue. Additionally, we also found that the malondialdehyde (MDA) content in the irradiation groups was elevated after the X‐ray treatment when compared to the untreated groups, suggesting that irradiation caused colon tissue injury. SeNPs pretreatment significantly decreased the expression of MDA induced by X‐ray in colon tissue (Figure 6C), which indicates the protection effects of SeNPs against X‐ray irradiation. X‐ray‐triggered colon tissue injury may promote the inflammation response. SeNPs pretreatment could maintain a higher selenium level in the blood and intestinal tissue (Figure S8, Supporting Information). Therefore, we asked wondered SeNPs treatment would endow the gut tissue to possess better resistance to X‐ray‐induced damages. To explore this inquiry, we investigated the inflammatory cytokines expression in SeNP‐treated mice with or without exposure to X‐ray. SeNPs pretreatment significantly inhibited the suppression effects on the anti‐inflammatory cytokine IL‐10 expression induced by X‐ray (Figure 6D). Additionally, compared with the untreated groups, X‐ray treatment increased the creation of pro‐inflammatory cytokines (Figure 6E–G), including IL‐6, TNF‐α, and IFN‐γ, which was evidenced by the reduced production of these cytokines as SeNPs pretreatment inhibited the inducing ability of X‐rays. To advance the examination of the inhibitory effect of SeNPs on X‐ray‐induced colitis, we examined the expression of STING and GPX4 in colon tissues aftertreatment (Figure 6H–M). Upregulated levels of STING and γH2AX were observed in the colon tissue (Figure 6H,I; Figure S9, Supporting Information) accompanied by the increased expression of inflammatory factors IL‐1β and IL‐6. However, SeNPs pretreatment restrained the inducing capacity of X‐ray and upregulated the manifestation of GPX4. In combination, these results demonstrate that SeNPs exhibits protective effects against X‐ray irradiation through inhibiting cGAS‐STING pathway activation.

### 2.7 SeNPs Regulate Gut Microbiota

**Figure 7:**
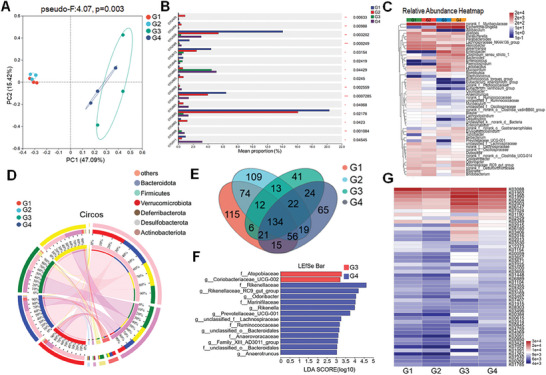
Effects of SeNPs and X‐ray on the gut microbiota abundances. A) The Bray Curtis PCoA combined with PERMANOVA analysis showed significant differences in the composition of gut bacterial communities between the irradiation group and the control group (n = 3, pseudo‐F: 4.07, PERMANOVAR). B) Significant inter‐group difference analysis of OUT levels in fecal samples from the Control, SeNPs, X‐ray, and SeNPs+X‐ray (one‐way ANOVA). C) Heat map of relative abundance of intestinal flora. D) The distribution of microbial species present in different microbial samples under different treatment groups. E) The Venn diagram illustrating the intestinal microbiota across various groups, wherein the numerical values shared between different circles denote the quantity of identical bacterial species. F) LDA identified a significant abundance of genera in different taxa. Groups that meet the LDA significance threshold of 2. The Venn diagram comparing intestinal flora among distinct groups depicts the quantity of shared bacterial species indicated by the values intersecting different circles. G) Distribution of KO assignments to analyze the individual gene in different samples by PICRUSt2 function. G1: Control, G2: SeNPs, G3: X‐ray, G4: X‐ray + SeNPs.

The microbiota serves as a vital component of regulating and training the gut immune system.^[^ ^34^ ^]^ Therefore, we also examined whether SeNPs treatment could affect the abundance of gut microbiota by using 16s sequencing technology. First, The analysis of Bray Curtis PCoA detection combined with PERMANOVA calculation indicated that there was a significant difference in the composition of intestinal bacterial communities between the irradiated and control groups at the entire OTU level, specifically manifested in the distance between the sample points of the irradiated and nonirradiated groups. SeNPs treatment intervention was able to narrow this difference. (**Figure** **7**A,B). It was found that the plenitude and variety of the intestinal microbiome decreased after irradiation, whereas oral administration of SeNPs increased the abundance and kinds of the intestinal microflora to a certain degree. A heat map analysis of the proportional prevalence (generic level) of the gut microbiota was performed on all samples from the different treatment groups (Figure 7C). The taxonomic associations among the microbial communities at the Phylum level were also evaluated. In comparison to the normal intestinal flora (group G1), X‐ray irradiation (group G3) reduced the bacterial abundance of Bacteroidota, Verrucomicrobiota, and Actinobacteriota (expressed in proportion to the size of the range). In contrast, SeNPs was able to increase the abundance of the above bacteria after irradiation, and had a similar abundance compared with the non‐irradiated group, indicating the protective effect of SeNPs on the intestinal microbiota after irradiation (Figure 7D). Related studies have reported that the intestinal flora of mice can be significantly changed by X‐ray irradiation. Venn diagram showed that SeNPs treatment made the intestinal microbiota of irradiated mice more similar to that of unirradiated and normal mice compared to irradiated mice (Figure 7E). Additionally, we studied different enriched taxa within different taxa by linear discriminant analysis (LDA) effect size analyses. The result shows the dominant taxa and their effects at different taxonomic levels from phylum to genus. The aboundance of beneficial bacteria was found in the mice that pretreated with SeNPs and then irradiated with X‐ray, when compared with X‐ray alone treatment. For example, *Prevotellaceae*, is well known as the intestinal symbionts. The reduction of *Prevotellaceae* may result in the exposure of bacterial endotoxin systems and increase intestinal permeability, which may trigger the imbalance of intestinal homeostasis. SeNPs pretreatment increases the abundance of *Prevotellaceae, Rikenellaceae*, and *Rikenella*, which suggests that SeNPs may help for increasing the abundance of beneficial bacteria upon radiation treatment (Figure 7F). Additionally, KEGG Orthology (KO) assignments demonstrate that X‐ray irradiation may alter the biological function of the microbial community. Under SeNPs treatment, the gene level is highly similar to that of the nonirradiated group, indicating that SeNPs may take part in preserving the normal function of the gut microbiota. For example, the iron complex transport system related genes such as K02015(iron complex transport system permease protein), K02016(iron complex transport system substrate‐binding protein) and K02013(iron complex transport system ATP‐binding protein) were upregulated upon X‐ray irradiation. X‐ray treatment may result in disruption of iron homeostasis and thus result in overproduction of harmful metabolites and affects downstream functions through specific signaling pathways.^[^ ^35^ ^]^ However, SeNPs pretreatment inhited the inducing capacity of X‐ray as indicated by the decreased level of these genes, which are simalar to the untreated groups. Evidence demonstrated that radiation could induce the imbalance of iron homeostasis and facilitate ferroptosis and result in colitis.^[^ ^5a^ ^]^ We found that SeNPs treatment could induce upregulation of GPX4 expression in the colon tissue, which may suggest that SeNPs inhibited radiation‐induced ferroptosis and the iron homeostasis disruption by inducing GPX4 expression and thus contributed to maintaining the gut microenvironment balance (Figure 7G). In conclusion, SeNPs could modulate the abundance of intestinal flora to maintain the host's microbial diversity and antagonize the intestinal inflammation induced by radiotherapy.

## 3 Conclusion

Radiation colitis is one of the most common complications in patients undergoing pelvic radiotherapy, and lack of effective treatment in the clinic. Finding effective strategies to combat radiation colitis is a topic of intense research. We found that the essential trace element Se holds promising application in reversing X‐ray‐induced intestinal injury and inflammation response. X‐ray irradiation could trigger a large amount of ROS to accumulate in intestinal epithelial cells, which induces DNA damage and facilitates the release of cytosolic DNA, thereby activating the cGAS‐STING‐IRF3/NF‐κB signaling pathway, which then contributes to the development of inflammation. However, Se, especially for SeNPs, treatment induced expression of selenoprotein, which endowed cells with higher antioxidative activity to restrict the oxidative stress and then block the inflammation signaling pathway transduction. Additionally, SeNPs pretreatment may also participate in maintaining the beneficial flora population, thus contributing to the maintenance of intestinal homeostasis. Taken together, the above results suggest that SeNPs play a regulatory role in restricting X‐ray‐induced intestinal tissue damage and the inflammation response with the involvement of increasing selenoprotein expression and decreasing DNA damage response as well as the cGAS‐STING pathway cascade, which may provide insight into the use of Se in radiation colitis treatment.

## 4 Experimental Section

### Materials

Selenocystine (SeCys_2_) and sodium selenite (Na_2_SeO_3_, SeIV) were acquired from Sigma–Aldrich (Shanghai, China). SeNPs were synthesized following the method outlined previously (Figure S1, Supporting Information).^[^ ^36^ ^]^ Bovine serum albumin (BSA), propidium iodide (PI), fetal bovine serum (FBS), phosphate‐buffered saline (PBS), DCF (H_2_DCFDA) probe, and MitoTracker Green were obtained from ThermoFisher Scientific (Shanghai, China). mPEG‐PCL was purchased from Merck Technology Co., Ltd., Ebselen from Macklin, M‐CSF from United Biotech, DMEM and IMDM media from Gibco. Antibodies were sourced from Cell Signaling Technology. All Elisa kits were obtained from BioLegend. All PCR primers were purchased from Sangon Biotech, and MPO was obtained from Beyotime Biotech Inc. The BCA protein assay kit (G3522) was from GBCBIO Technologies (Guangzhou, China). Anti‐CD44‐FITC (561859) and anti‐FoxP3‐FITC (13353442), Anti‐CD16/32‐PE (101307), anti‐CD4‐APC/Cy7 (B361057), anti‐CD25‐PE (B349987), anti‐CD11b‐FITC (B333716), anti‐Ly6G‐APC (B296099), Anti‐Ly6C‐PECy7 (2210385) were purchased from BioLegend.

### Cytotoxicity Evaluation

The IEC‐6 rat intestinal epithelial cell line, human normal colon epithelial cells NCM‐460, and rodent monocytic RAW264.7 macrophage leukemia cells were from the American Type Culture Collection (ATCC), located in Manassas, Virginia, USA. Cells were subjected to treatment with specific concentrations of different forms of Se for indicated times, and then cell viability was assayed by MTT. For the protective effects of Se against X‐ray, cells were pre‐exposed to Se for 6 h before undergoing X‐ray irradiation, followed by a 48 h culture period. After that cell viability was assessed by MTT.

### Clonogenic Assay

NCM‐460 cells (500 cells per well, 24 well plates) treated with Se (SeNPs, SeCys_2_, Ebselen, Na_2_SeO_3_, D‐SeCys_2_, D‐Ebselen) in the presence of X‐ray or not overnight, then replaced with fresh medium and cocultured for 14 days. After discarding the medium and washed with PBS three times, cells were methanol‐fixed and stained with crystal violet (0.5%, in 95% ethanol). Cell colonies were captured using a multi‐function detection system (Gene 5.0, Biotek).

### Cellular Localization and Uptake

To evaluate the cellular localization of SeNPs, the nanoparticles were loaded with coumarin‐6 (SeNPs‐coumarin‐6). After dialysis, SeNPs‐coumarin‐6 (2 µM) was added to IEC‐6 cells that were pretrained with lysotracker, and the distribution of nanoparticles was measured by fluorescence microscopy (Nikon Corporation, ECLIPSE Ti2‐E) at different incubation time. The cellular uptake of SeNPs was examined as previously described.^[^ ^37^ ^]^ Briefly, IEC‐6 cells were first pretreated with different endocytosis inhibitors for 1 h. SeNPs‐coumarin‐6 was then added and incubated for indicated times. For energy energy‐related uptake mechanism, cells were placed at 4 °C for 4 h followed by different treatments. The fluorescence intensity was recorded at the end of the experiments by using a fluorescence microplate reader (Cytation 5, Bio‐TekInstruments, Inc. Winooski, VT, USA).

### Assessment of Mitochondrial Membrane Potential

Flow cytometry was used to examine the mitochondrial membrane potential. In brief, cells were treated with Se and X‐ray for indicated times, then collected and stained with JC‐1 (10 µg mL^−1^) for 30 min at room temperature. Subsequently, cells were rinsed thrice with PBS and analyzed with a flow cytometer (Cytoflex, Beckman). At least 10000 cells were gathered for further studies.

### Analysis of Reactive Oxygen Species (ROS)

H_2_DCFDA sensor was used to assess the intracellular ROS levels. IEC‐6 and NCM‐460 cells (3 × 10^5^ cells mL^−1^ per well) were placed in contact with H_2_DCFDA (4 µM) at 37 °C for 30 min. Cells underwent pretreatment with Se for indicated times and then were rinsed thrice using PBS. After that, the cells were irradiated with or without X‐ray (16 Gy) and the fluorescence intensity was assessed utilizing a fluorescence microplate reader (Cytation 5, Bio‐Tek Instruments, Inc., Winooski, VT, USA) with excitation and emission wavelengths of 488 and 525 nm, respectively.

### Immunofluorescence

Cells were received with indicated treatments and then treated with fixative 4% polyoxymethylene glycol for 30 min and permeabilized with 0.1% Triton X‐100 solution. After the blockage with 5% BSA for 2 h, cells underwent incubation with phospho‐histone H2AX antibody (Ser139) at 4 °C overnight. The phospho‐histone H2AX expression was examined using a fluorescence microscope after the incubation with Alexa Fluor 488‐labeled Goat Anti‐Rabbit IgG for 1 h at room temperature.

### ELISA Analysis

Cytokine expression including TNF‐α, IL‐6, IL‐10, and IFN‐γ was examined by ELISA as per the instructions. IEC‐6 and RAW264.7 cells were received with the specified treatment of Se with or without X‐ray for indicated times. Then the supernatants were collected and subjected to an ELISA assay.

### Quantitative Reverse Transcription PCR

IEC‐6 cells received pre‐exposure to Se and then received with X‐ray irradiation and cultured for indicated times. Then the total RNA was extracted with Trizol reagent (Takara, Japan). After quantification, RNA was exposed to reverse transcription to obtain cDNA. The expression of selenoprotein, cGAS, STING, TBK1, TNF‐α, and IL‐6 were examined by CFX Connect Real‐Time PCR Detection System (Bio‐Rad, USA) using 2 × SYBR Green Master Mix (Bimake). GAPDH served as the internal control.

### Western Blotting Assay

The expression of the indicated protein was assessed using a western blotting assay as described previously. Briefly, cells were treated with indicated treatment for indicated times and then lysed with RIPA lysis buffer. The total protein was obtained via centrifugation and subjected to protein content determination. The protein (60 µg) was segregated by SDS‐PAGE electrophoresis and then relocated onto a polyvinylidene fluoride (PVDF) membrane (Millipore). After overnight incubation with primary antibody (diluted 1:1000) at 4 °C and then with secondary antibody conjugated to protein (1:3000) for 1 h at room temperature, protein expression was detected by X‐ray film exposure after the addition of enhanced chemiluminescence.

### X‐Ray‐Induced Colitis

All animal experiments were authorized by the Animal Experiment Ethics Committee of Jinan University. The C57BL/6J mice (female, 4–5 weeks old) were from the Animal Breeding Center of Jinan University (Guangzhou, China) (The animal ethical number: IACUC‐20230907‐01). The animals were kept in a specific pathogen‐free (SPF) environment for 7 days, and were divided into groups randomly in four divisions (n = 10): Control (G1), SeNPs (G2), X‐ray (G3), and X‐ray + SeNPs (G4). Mice in SeNPs and the combined treatment groups were pretreated with SeNPs (2 mg kg^−1^) every day for two weeks and then received with or without a single dose of X‐ray (16 Gy) for local abdominal irradiation on the first day, which is an effective strategy to induce radiation‐induced colitis.^[^ ^5^, ^37^ ^]^ After irradiation, the weight of mice was monitored each day, and the daily disease activity index (DAI) was calculated. At the culmination of the treatment, the mice were euthanased, and organs including the heart, liver, spleen, lungs, kidneys, and colon were collected for subsequent analyses.

### Flow Cytometry Analysis

Spleen, mesenteric lymph node, and colon tissue samples from the aforementioned animal experiments were collected and then digested with collagenase/hyaluronidase and DNase followed by homogenized to obtain a single cells suspension. Cells (1 × 10^7^ cells mL^−1^) were incubated with Mouse TruStain FcX and then stained with specific antibodies for 30 min at room temperature. After rinsing 3 times with PBS, cells were collected and assessed by Flow cytometry assay. The proportion of cells was gated according to the specific markers. For instance, CD45^+^CD11b^+^Gr1^+^ and CD45^+^CD11b^+^F4/80^+^CD206^+^ positive cells were denoted as MDSCs and M2 macrophages. For analysis, at least 10000 cells were gathered.

### H&E Staining and Immunofluorescence

Histopathological alterations in colon samples were assessed using H&E staining following the previously reported.^[^ ^38^ ^]^ Changes in the expression of inflammatory cytokines, STING, and GPX4 were examined by immunofluorescence assay. Briefly, after antigen retrieval, penetration, and blocking (1% BSA, dissolved in PBST (PBS + 0.1% Tween 20)), the colon tissues were raised in specific primary antibodies at 4 °C overnight. After three rinses with PBS, the tissue was stained for 30 min without exposure to light using secondary antibodies labeled with Alexa Fluor. The Hoechst 33342 dye was applied to the cell nucleus. In the end, expressions of these molecules were examined by a fluorescence microscope.

### Microbiome Analysis

The influence of SeNPs on the intestinal microbiota of mice after irradiation was detected by 16s rRNA gene sequencing and analysis (Shanghai Mejisi Biomedical Technology Co., Ltd.) was studied using the online platform of Majorbio Cloud Platform (www.majorbio.com). C57BL/6J female mice were orally administered for two weeks, followed by intraperitoneal irradiation according to the above method. During days 5–8 of treatment, mouse feces were collected and stored in a −80 °C freezer for subsequent gene sequencing. Microbial community genomic DNA was extracted with the E.Z.N.A. soil DNA kit (Omega Bio‐tek, Norcross, GA, U.S.), and the quality of the extracted genomic DNA was checked with 1% agarose gel electrophoresis, while DNA concentration and purity were investigated using NanoDrop2000 (Thermo Scientific, USA). Subsequently, the extracted DNA was used to generate 16S rRNA libraries (V3‐V4 region), and community analysis was determined using the Illumina PE300 sequencing platform. A short fragment library was constructed based on the characteristics of the amplified 16S region, and the library was pairwise sequenced on the Illumina PE300 sequencing platform. OTUs were clustered through reads assembly and filtering, followed by species annotation and abundance analysis. Alpha Diversity and Beta Diversity analyses not only revealed differences in species composition and community structure between samples but also allowed for personalized analysis and deep data mining as required by the project. The representative sequences of OTUs were classified and evaluated using mothur software. Finally, all data were analyzed on the Mejisi Biomedical Cloud Platform.

### Statistical Analysis

The experiments were performed in triplicate, and the data are depicted as mean ± standard deviation (mean ± SD). Statistical analyses for all data were analyzed by using an unpaired Student's t‐test (normal distribution) or One‐way ANOVA methods using GraphPad Prism software (version 10.1.2). *P < 0.05, **P < 0.01, ***P < 0.001, and ****P < 0.0001 are denoted as significant differences between the comparing groups.

## Conflict of Interest

The authors declare no conflict of interest.

## Supporting information



Supporting Information

## Data Availability

The data that support the findings of this study are available from the corresponding author upon reasonable request.

## References

[1] a) M. Al‐Sarraf , M. LeBlanc , P. Giri , K. K. Fu , J. Cooper , T. Vuong , A. A. Forastiere , G. Adams , W. A. Sakr , D. E. Schuller , J. Clin. Oncol. 2023, 41, 3965; 37586209 10.1200/JCO.22.02764

[2] a) L. J. Wedlake , Proc. Nutr. Soc. 2018, 77, 357; 29607792 10.1017/S0029665118000101

[3] a) K. Wang , J. E. Tepper , CA Cancer J. Clin. 2021, 71, 437; 34255347 10.3322/caac.21689

[4] N. K. Cleveland , J. Torres , D. T. Rubin , Gastroenterology 2022, 162, 1396. 35101421 10.1053/j.gastro.2022.01.023

[5] a) Y. Feng , X. Luo , Z. Li , X. Fan , Y. Wang , R.‐R. He , M. Liu , Nat. Commun. 2023, 14, 5083; 37607944 10.1038/s41467-023-40794-w PMC10444825

[6] L. Liu , Y. Wang , S. Yu , H. Liu , Y. Li , S. Hua , Y. G. Chen , Adv. Sci. 2023, 10, 2300708. 10.1002/advs.202300708 PMC10427365 37261975

[7] J. Wang , C.‐Y. Chang , X. Yang , F. Zhou , J. Liu , J. Bargonetti , L. Zhang , P. Xie , Z. Feng , W. Hu , Nat. Commun. 2024, 15, 137. 38167344 10.1038/s41467-023-44390-w PMC10762193

[8] J. Xie , C. Wang , N. Wang , S. Zhu , L. Mei , X. Zhang , Y. Yong , L. Li , C. Chen , C. Huang , Biomaterials 2020, 244, 119940. 32200103 10.1016/j.biomaterials.2020.119940

[9] J. F. Burgueño , M. T. Abreu , Nat. Rev. Gastroenterol. Hepatol. 2020, 17, 263. 32103203 10.1038/s41575-019-0261-4

[10] a) P. Garred , A. J. Tenner , T. E. Mollnes , Pharmacol. Rev. 2021, 73, 792; 33687995 10.1124/pharmrev.120.000072 PMC7956994

[11] a) C. Chen , P. Xu , Trends Cell Biol. 2023, 33, 630; 36437149 10.1016/j.tcb.2022.11.001

[12] a) E. Vringer , S. W. Tait , Cell Death Differ. 2023, 30, 304; 36447047 10.1038/s41418-022-01094-w PMC9950460

[13] a) H. Maekawa , T. Inoue , H. Ouchi , T.‐M. Jao , R. Inoue , H. Nishi , R. Fujii , F. Ishidate , T. Tanaka , Y. Tanaka , Cell Rep. 2019, 29, 1261; 31665638 10.1016/j.celrep.2019.09.050

[14] A. Ablasser , Z. J. Chen , Science 2019, 363, eaat8657. 30846571 10.1126/science.aat8657

[15] a) J. Zheng , J. Mo , T. Zhu , W. Zhuo , Y. Yi , S. Hu , J. Yin , W. Zhang , H. Zhou , Z. Liu , Mol. Cancer 2020, 19, 133; 32854711 10.1186/s12943-020-01250-1 PMC7450153

[16] a) W. Wang , B. Cui , Y. Nie , L. Sun , F. Zhang , Protein & Cell 2024, 15, 83; 37470727 10.1093/procel/pwad044 PMC10833463

[17] I. Moraitis , J. Guiu , J. Rubert , Trends Endocrinol. Metab. 2023, 34, 489. 37336645 10.1016/j.tem.2023.05.006

[18] a) B. Wang , X. Chen , Z. Chen , H. Xiao , J. Dong , Y. Li , X. Zeng , J. Liu , G. Wan , S. Fan , Exp. Mol. Med. 2023, 55, 55; 36599931 10.1038/s12276-022-00911-z PMC9898499

[19] a) Q. Huang , Z. Liu , Y. Yang , Y. Yang , T. Huang , Y. Hong , J. Zhang , Q. Chen , T. Zhao , Z. Xiao , Adv. Sci. 2023, 10, 2300880; 10.1002/advs.202300880 PMC10323656 37408520

[20] a) M. P. Rayman , Lancet 2012, 379, 1256; 22381456 10.1016/S0140-6736(11)61452-9

[21] S. Li , W. Sun , K. Zhang , J. Zhu , X. Jia , X. Guo , Q. Zhao , C. Tang , J. Yin , J. Zhang , J. Anim. Sci. Biotechnol. 2021, 12, 1. 33993883 10.1186/s40104-021-00587-x PMC8127211

[22] S. Massironi , C. Viganò , A. Palermo , L. Pirola , G. Mulinacci , M. Allocca , L. Peyrin‐Biroulet , S. Danese , Lancet Gastroenterol Hepatol 2023, 8, 579. 36933563 10.1016/S2468-1253(23)00011-0

[23] H. Liu , J. Shen , W. Liu , D. Liu , L. Cheng , B. Huang , Immunobiology 2024, 229, 152839. 39094396 10.1016/j.imbio.2024.152839

[24] R. S. Esworthy , J. H. Doroshow , F.‐F. Chu , Free Radical Biol. Med. 2022, 188, 419. 35803440 10.1016/j.freeradbiomed.2022.06.232 PMC9341242

[25] Q. Guo , X. Xu , X. Lai , J. Duan , D. Yan , D. Wang , Exploration 2024, 20230115, 10.1002/EXP.20230115. PMC12087400 40395757

[26] D. Zhang , L. Chen , Y. Zhao , H. Ni , Q. Quan , J. Ma , L. Guo , Cancer Nanotechnol. 2024, 15, 25.

[27] a) W. Sang , L. Xie , G. Wang , J. Li , Z. Zhang , B. Li , S. Guo , C. X. Deng , Y. Dai , Adv. Sci. 2021, 8, 2003338; 10.1002/advs.202003338 PMC7887592 33643804

[28] B. Feng , Y. Zhang , T. Liu , L. Chan , T. Chen , J. Zhao , Chin. Chem. Lett. 2023, 34, 108264.

[29] Y. Jin , D. Li , X. Zheng , M. Gao , W. Wang , X. Zhang , W. Kang , C. Zhang , S. Wu , R. Dai , Adv. Sci. 2024, 11, 2308905. 10.1002/advs.202308905 PMC11077689 38419379

[30] J. Tan , M. Wang , B. Ding , J. Lin , Coord. Chem. Rev. 2023, 493, 215316.

[31] P. Li , Q. Ou , S. Shi , C. Shao , Cell. Mol. Immunol. 2023, 20, 558. 36973490 10.1038/s41423-023-00998-y PMC10040934

[32] J. Li , Y. Wang , W. Shen , Z. Zhang , Z. Su , X. Guo , P. Pei , L. Hu , T. Liu , K. Yang , Adv. Sci. 2024, 11, 2400845. 10.1002/advs.202400845 PMC11095197 38520732

[33] S. Chen , A. F. Saeed , Q. Liu , Q. Jiang , H. Xu , G. G. Xiao , L. Rao , Y. Duo , Sign. Transduct. Targeted Ther. 2023, 8, 207. 10.1038/s41392-023-01452-1 PMC10200802 37211559

[34] a) O. Koren , L. Konnikova , P. Brodin , I. U. Mysorekar , M. C. Collado , Nat. Rev. Gastroenterol. Hepatol. 2024, 21, 35; 38097774 10.1038/s41575-023-00864-2 PMC12635954

[35] L. Xiao , R. Tang , J. Wang , D. Wan , Y. Yin , L. Xie , Sci. China Life Sci. 2023, 66, 1952. 37515687 10.1007/s11427-022-2302-5

[36] a) T. Liu , L. Xu , L. He , J. Zhao , Z. Zhang , Q. Chen , T. Chen , Nano Today 2020, 35, 100975;

[37] H. Lai , X. Zhang , Z. Song , Z. Yuan , L. He , T. Chen , Chem. Eng. J. 2020, 391, 123563.

[38] J. Ouyang , B. Deng , B. Zou , Y. Li , Q. Bu , Y. Tian , M. Chen , W. Chen , N. Kong , T. Chen , J. Am. Chem. Soc. 2023, 145, 12193. 37208802 10.1021/jacs.3c02179

