# Supplementary material for: Selenium Attenuates Radiation Colitis by Regulating cGAS‐STING Signaling
Source: Adv Sci (Weinh). 2024 Sep 30;11(44):2403918. doi: 10.1002/advs.202403918 (PMC11600249; doi:10.1002/advs.202403918)
Supplement: Supplementary file 1 — Supporting Information [file ADVS-11-2403918-s001.docx]

**Supporting Information**

**Selenium Attenuates Radiation Colitis by Regulating cGAS-STING Signaling**

Qian Xue#, Haoqiang Lai#, Haimei Zhang#, Guizhen Li, Fen Pi, Qifeng Wu, Siwei Liu, Fang Yang* and Tianfeng Chen*

Department of Chemistry, State Key Laboratory of Bioactive Molecules and Druggalibility Assessment, Laboratory of Viral Pathogenesis & Infection Prevention and Control of Ministry of Education, MOE Key Laboratory of Tumor Molecular Biology, Jinan University, Guangzhou, 510632, China.

# These authors contribute equally to this work

* Corresponding authors.

E-mail: tyoung@jnu.edu.cn (Prof. F. Yang), tchentf@jnu.edu.cn (Prof. T. Chen)

**Supporting Figures**


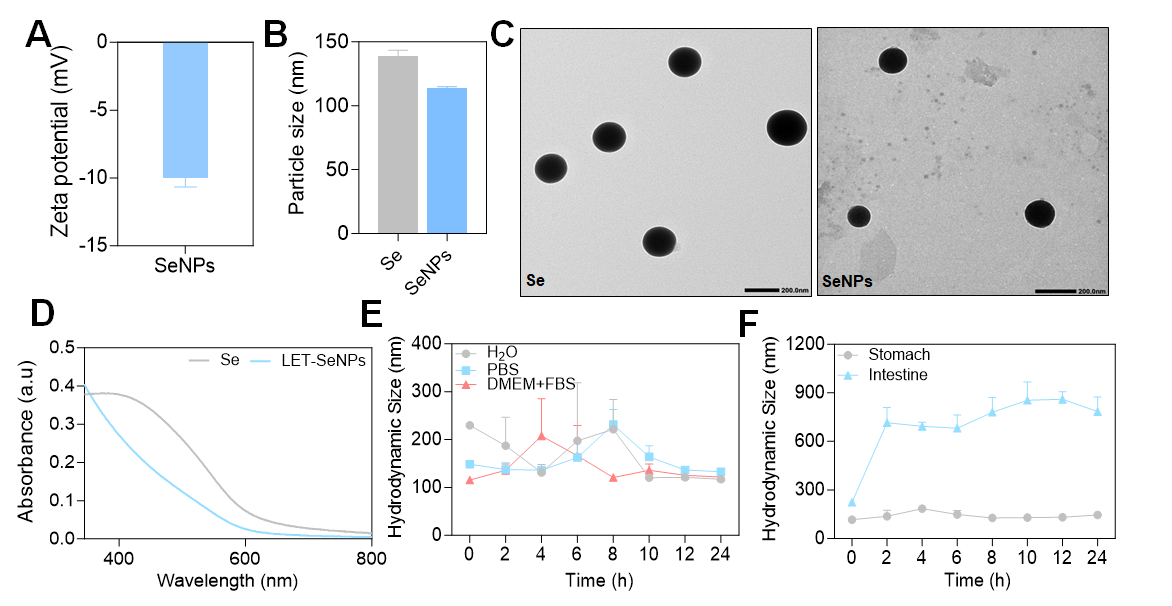


**Figure S1. Characterization of SeNPs.** The Zeta potential **A**), particle size **B**) and TEM of lentinan modified SeNPs **C**). The ultraviolet analysis **D**), stability in different condition **E**) and in the gastroenteric fluid simulation environment of lentinan modified SeNPs **F**). Data were expressed as mean ± SD, n = 3.


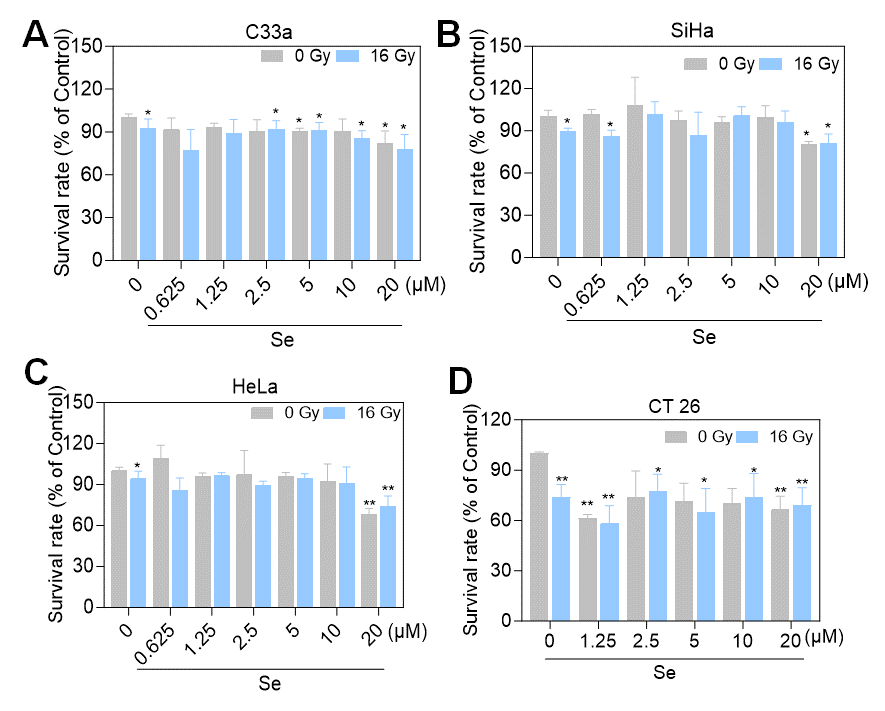


**Figure S2. Effects of SeNPs and X-ray on the cell viability of cervical cancer cell lines and CT26 cells.** The cell viability of C33a A), Siha B), Hela C) and CT26 cells D) after the treatment of SeNPs and X-ray. Cells were pretreated with indicated concentration of SeNPs for 6 h and then exposure to X-ray (16 Gy). After incubation for 72 h, the cell viability was examined by MTT assay. Data are represented as mean ± SD, n = 4. **P* < 0.05 and ** *P* < 0.01 are considered as statistically significant differences with the control groups.


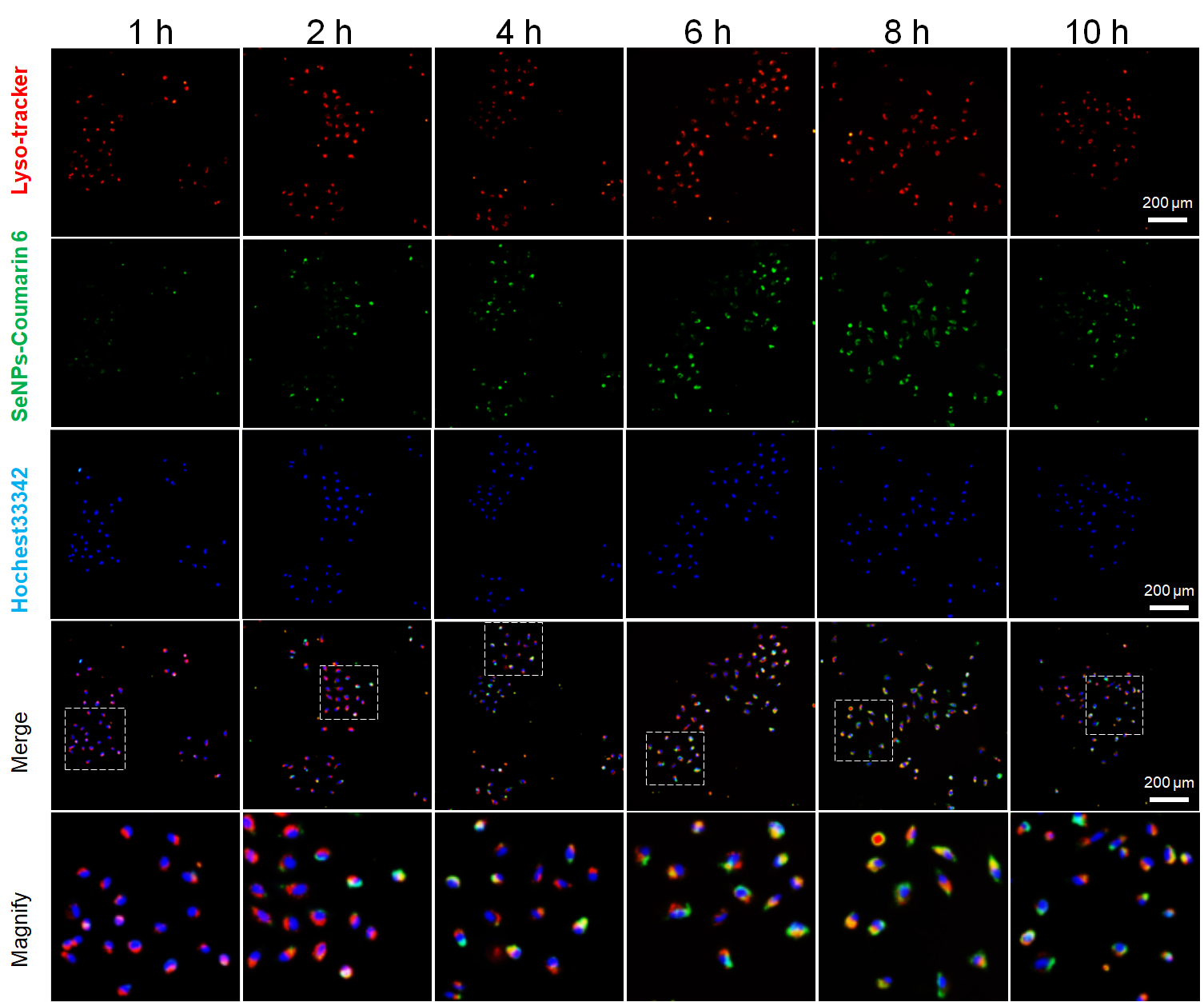
**Figure S3. The intracellular localization of SeNPs in IEC-6 cells.** To monitor the intracellular localization of the nanoparticles, SeNPs were loaded with coumarin 6. Cells were prestained with lysotracker and then treated with 2 μM SeNPs and the intracellular localization of SeNPs was observed by fluorescence microscope.


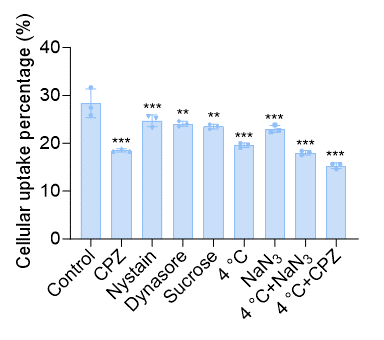


**Figure S4. The cellular uptake of SeNPs under different endocytosis inhibitor treatments in IEC-6 cells.** Data are represented as mean ± SD, n = 3. ***P* < 0.001 and ****P* < 0.0001 are considered as statistically significant differences.


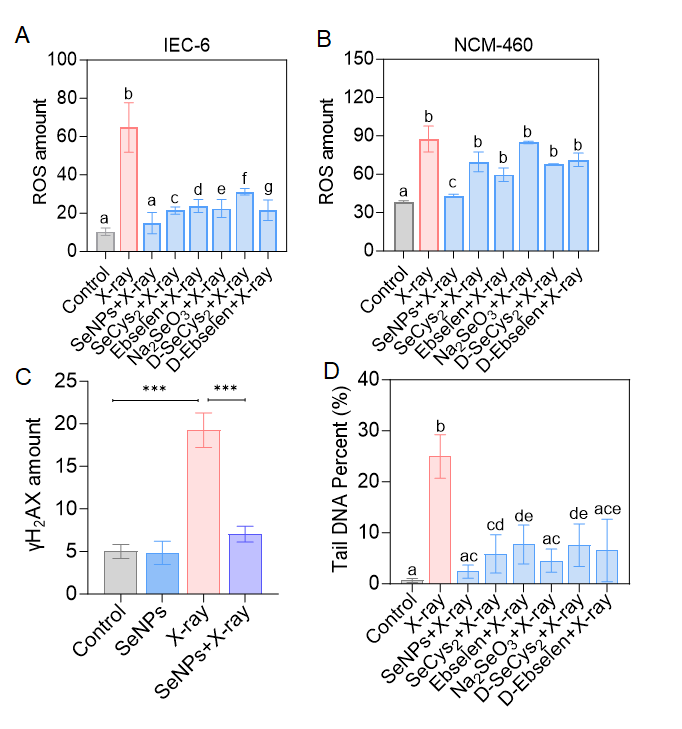


**Figure S5. Quantification of the fluorescence intensity of DCF in different treatment groups in IEC-6 cells (A), NCM-460 cells (B), and γH_2_AX expression in IEC-6 cells (C).** Data are represented as mean ± SD, n = 3. (D) Quantification of the tail DNA after different treatment. Data are represented as mean ± SD, n = 12. Characters a-g are represented as significant differences with different treatment group. *** *P* < 0.01 is considered as significant difference. Bar with a, b, c, d and e are denoted as significant differences with the *P* < 0.05.


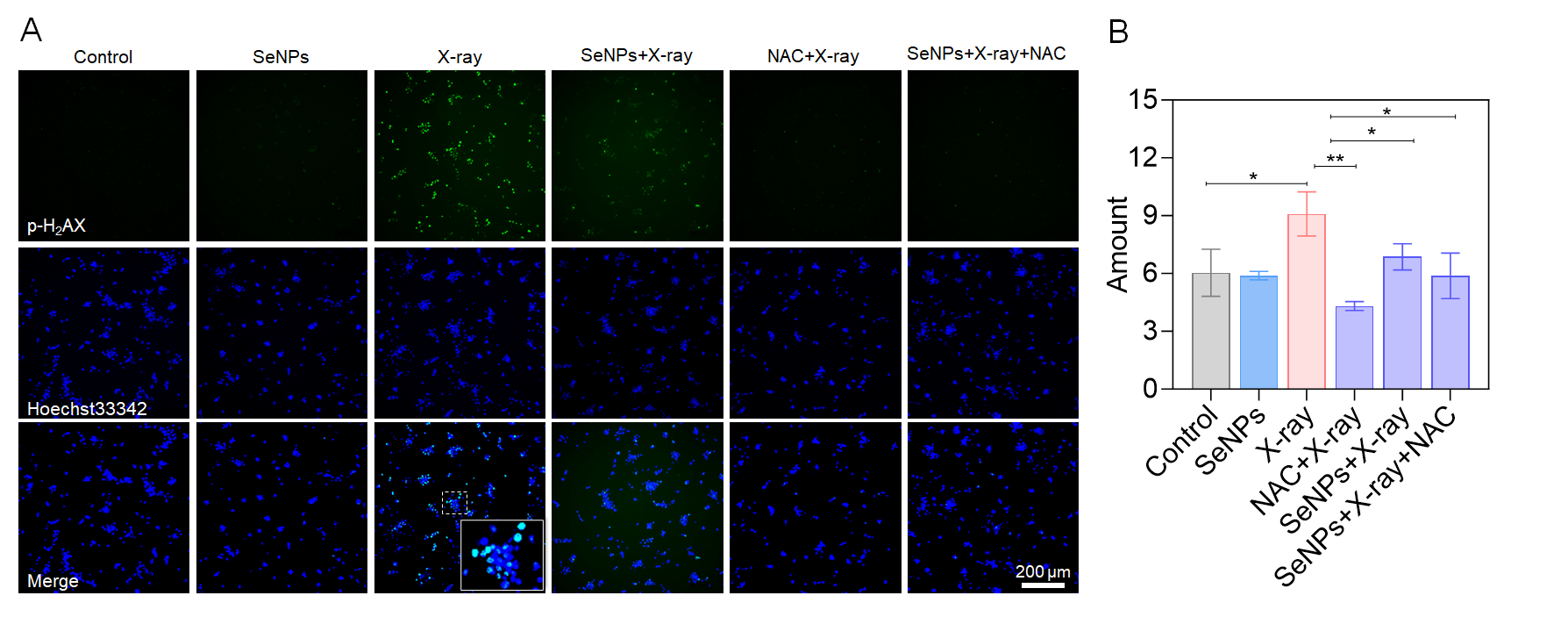


**Figure S6. The expression of** **γH2AX under different treatments.** (A) Representative images of the expression of γH2AX in IEC-6 cells. (B) Quantification of the expression of γH2AX in different groups. Data are represented as mean ± SD, n = 3. * *P* < 0.05, ** *P* < 0.01, respectively.


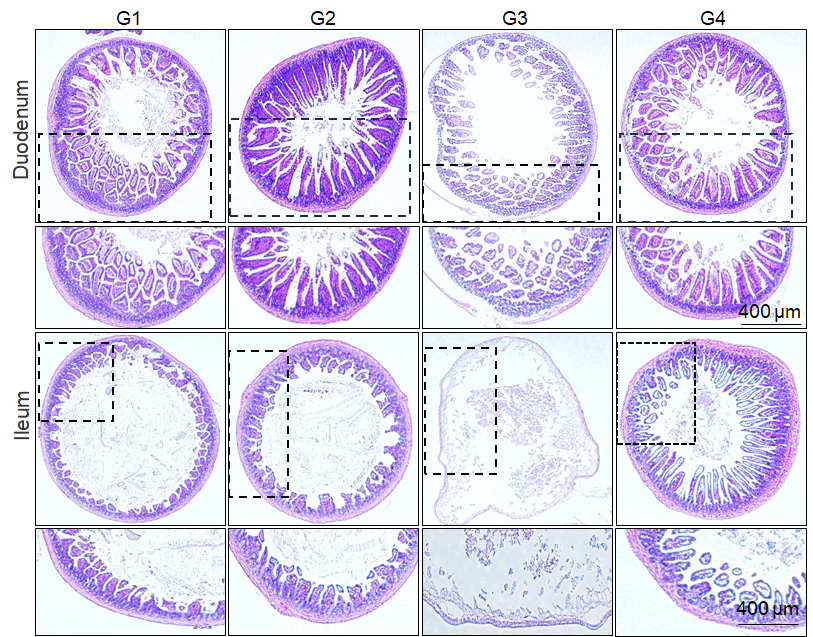


**Figure S7. Histological changes of the duodenum and ileum tissues after different treatments.** G1: Control, G2: SeNPs, G3: X-ray, G4: X-ray + SeNPs.


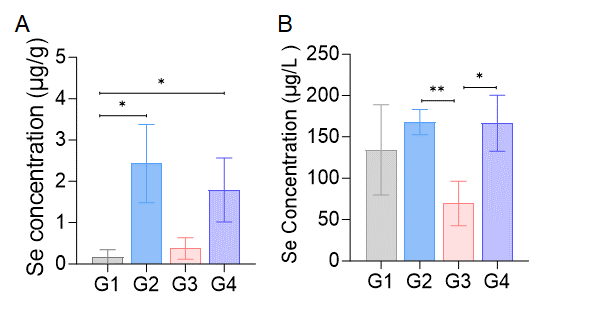


**Figure S8. The selenium content in the intestinal tissues (A) and blood (B).** G1: Control, G2: SeNPs, G3: X-ray, G4: X-ray + SeNPs. Data are represented as mean ± SD, n = 3. **P* < 0.05 and ***P* < 0.01 are considered as statistically significant differences.


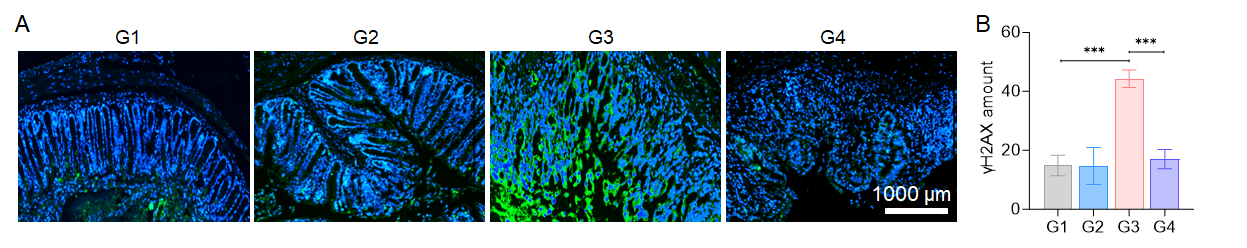


**Figure S9. The images of the γH2AX expression in the intestinal tissues in different treatment groups (A) and the quantification of the expression of γH2AX (B). G1**: Control, G2: SeNPs, G3: X-ray, G4: X-ray + SeNPs. Data are represented as mean ± SD, n = 3. ****P* < 0.001 is considered as statistically significant difference.
